# Impacto da Impressão em 3D nas Cirurgias de Cardiopatias Congênitas: Revisão Sistemática e Metanálise

**DOI:** 10.36660/abc.20240430

**Published:** 2024-11-27

**Authors:** Davi Shunji Yahiro, Mariana de Paula Cruz, Brenda Ficheira Coelho Ribeiro, Luiza Meireles Teixeira, Maria Fernanda Ribeiro Mendes de Oliveira, Aurea Lúcia Alves de Azevedo Grippa de Souza, Ana Flávia Malheiros Torbey, Juliana Serafim da Silveira, Claudio Tinoco Mesquita

**Affiliations:** 1 Universidade Federal Fluminense Niterói RJ Brasil Universidade Federal Fluminense, Niterói, RJ – Brasil; 2 Pró-Cardíaco Hospital Rio de Janeiro RJ Brasil Pró-Cardíaco Hospital, Rio de Janeiro, RJ – Brasil

**Keywords:** Impressão Tridimensional, Cardiopatias Congênitas, Cuidados Pré-Operatórios, Medicina de Precisão

## Abstract

**Fundamento:**

As cardiopatias congênitas (CCs) impõem um desafio importante ao tratamento cirúrgico devido à complexidade da anatomia cardíaca. A impressão em 3D surgiu como uma ferramenta promissora no planejamento perioperatório, no direcionamento intraoperatório, e na educação médica em cirurgia de CC.

**Objetivo:**

Avaliar sistematicamente a literatura sobre o uso e os benefícios da tecnologia de impressão em 3D nas intervenções cirúrgicas de CCs.

**Métodos:**

Realizou-se uma busca sistemática nas bases de dados PubMed e EMBASE por estudos publicados até fevereiro de 2024. Incluímos estudos controlados e não controlados investigando o papel da impressão em 3D em cirurgias em pacientes com CCs. Conduzimos uma metanálise de braço único estimando a proporção de mudança no planejamento do tratamento devido ao uso de modelos impressos em 3D. Além disso, estudos que compararam o uso de impressão em 3D com tratamento convencional foram incluídos na metanálise. Um valor de p<0,05 foi considerado estatisticamente significativo.

**Resultados:**

Um total de 21 estudos preencheram os critérios de inclusão, incluindo 444 pacientes submetidos a cirurgias de CCs com auxílio da impressão em 3D. O planejamento pré-operatório com ajuda de modelos 3D levou a mudança de decisões cirúrgicas em 35 dos 75 casos (51,8%; IC95% 26,6-77,0%, I2=80,68%, p=0,001) e redução no tempo total da cirurgia em 22,25 minutos a favor da impressão e 3D (IC95% - 49,95; 5,80 min; I2=0%; p=0,817), mas sem significância estatística. Embora em uma amostra menor, outros desfechos (ventilação mecânica e tempo na unidade de terapia intensiva) demonstraram algum benefício da tecnologia mas sem significância estatística.

**Conclusões:**

Ao fornecer modelos anatômicos personalizados, a impressão em 3D pode facilitar o planejamento e a execução da cirurgia. Mais estudos são necessários para investigar os efeitos da impressão em 3D na redução nos tempos da intervenção, de internação e de ventilação mecânica.

## Graphical abstract

**Figura Central:**
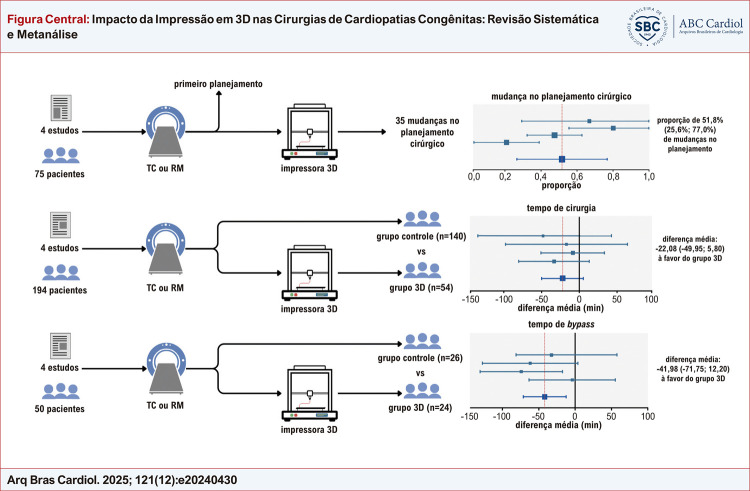
: Impacto da Impressão em 3D nas Cirurgias de Cardiopatias Congênitas: Revisão Sistemática e Metanálise

## Introdução

As anomalias cardíacas são as malformações congênitas mais comuns em nascidos vivos no mundo. No Brasil, estima-se que aproximadamente 25 757 novos casos ocorram por ano, e a região sudeste apresenta a maior prevalência, com 10 novos casos/1000 nascidos vivos. No entanto, este número pode ser ainda maior devido à subnotificação dos casos de doença cardíaca congênita.^1,2^

Em muitas cardiopatias congênitas (CCs), é necessária uma análise abrangente para o diagnóstico e o tratamento corretos, uma vez que elas podem combinar defeitos, aumentando a complexidade de cada caso. Tal fato requer um tratamento individualizado e multidisciplinar, dependendo da sua complexidade. Para planejar uma intervenção, é necessário examinar meticulosamente a anatomia das estruturas. Uma metanálise demonstrou que o modelo tridimensional (3D) de uma CC apresentou um desvio padrão de 0,04 mm, IC (-0,16; 0,23) em comparação a imagens médicas digitais.^3^ Portanto, a modelagem e a impressão de modelos 3D de imagens médicas podem fornecer uma visualização auxiliar da anomalia específica.

Modelos 3D de qualquer condição médica são possíveis por exames de imagens. A tomografia computadorizada (TC) e a ressonância magnética (RM) são as técnicas mais confiáveis para obtenção de dados para a construção de modelos anatômicos. Antes da impressão, as imagens precisam ser transformadas em um modelo digital e divididas em camadas finas para serem reconstruídas em uma impressora 3D, formando a peça final. Nos últimos anos, a impressão 3D surgiu como uma tecnologia proeminente no campo da medicina, oferecendo aplicações versáteis. Sua utilização varia desde o planejamento cirúrgico, fins educacionais, até estratégias de comunicação efetiva. A natureza multifacetada da impressão 3D promoveu um avanço significativo nas práticas clínicas, permitindo maior precisão e eficiência, e melhores desfechos.^4^

Apesar do potencial promissor da tecnologia da impressão 3D em melhorar o planejamento e a execução de cirurgias cardíacas, particularmente em pacientes com CCs, existe uma clara ausência de dados robustos nessa área. Essa falta de dados abrangentes e de alta qualidade limita nosso entendimento da real aplicabilidade e do verdadeiro impacto da impressão 3D nesse contexto. Enquanto estudos preliminares e supostas evidências sugerem que modelos cardíacos construídos por impressão 3D poderiam fornecer melhores *insights* no pré-operatório e possivelmente melhorar os desfechos cirúrgicos, a necessidade de uma pesquisa sistemática, em grande escala, é evidente. Esses estudos ajudariam a quantificar os benefícios, otimizar o uso dessa tecnologia, e validar sua eficácia e custo-efetividade no âmbito clínico. Até lá, o potencial máximo da impressão 3D no planejamento de cirurgias cardíacas continua pouco explorado e subutilizado. Dada a emergência no ambiente médico, algumas pesquisas sistemáticas avaliaram o impacto da impressão 3D nas condições cardiovasculares.^3-6^ No entanto, nenhuma delas teve como foco a CC ou a intervenção cirúrgica. Nesse sentido, este estudo tem como objetivo avaliar e analisar as aplicações atuais da impressão 3D nas intervenções cirúrgicas nas CCs.

## Métodos

### Fonte de dados e estratégia de busca

Uma busca sistemática da literatura foi conduzida nos bancos de dados eletrônicos incluindo PubMed e Embase. A estratégia de busca usou uma combinação de palavras e termos relacionados à impressão 3D, CCs, cirurgia e intervenções. Operadores booleanos e filtros de busca foram aplicados para assegurar uma cobertura abrangente da literatura relevante. A busca bibliográfica foi conduzida em fevereiro de 2024, contendo todas as palavras publicadas até aquela data. A estratégia completa da pesquisa bibliográfica encontra-se no Apêndice 1. Além disso, realizou-se uma busca manual de listas de referências para identificar outros estudos que possam não ter sido identificados nas buscas eletrônicas.

### Critérios de elegibilidade e processo de seleção

Dois revisores independentes (DY e MC) rastrearam títulos e resumos das citações obtidas para identificar estudos elegíveis. Artigos completos foram então avaliados quanto à elegibilidade com base nos critérios de inclusão e exclusão pré-definidos. Os estudos foram incluídos se tivessem avaliado a utilidade da impressão 3D no planejamento, desempenho ou prognóstico de CCs. Estudos de revisão, metanálises, carta, estudos experimentais e séries de casos com menos de cinco participantes foram excluídos da análise. Qualquer discrepância entre revisores foi resolvida por discussão ou consulta om um terceiro revisor.

### Extração dos dados

Os dados foram extraídos de maneira independente por dois revisores (DY e MC), usando um formulário padronizado. Os dados extraídos incluíram delineamento do estudo, tamanho amostral, modelo da impressora 3D, custo, método de imagem, *software* de segmentação, material de impressão, propósito do estudo, condição no tratamento, achados principais, como mudança no plano cirúrgico ou tempo de cirurgia, e desfechos secundários: tempo de *bypass*, ventilação mecânica, e tempo de internação. Além disso, coletamos informações sobre mudança na decisão ou no tempo cirúrgicos quando disponíveis. Discordâncias entre os revisores foram resolvidas por consenso ou consulta com um terceiro revisor.

### Avaliação do risco de viés

A qualidade dos artigos incluídos foi avaliada por dois autores (MC e LT) usando duas ferramentas diferentes de avaliação de risco de viés – o *JBI Critical Appraisal Checklist for Case Series* para estudos retrospectivos sem um grupo para comparação e a ferramenta ROBINS-I para estudos prospectivos não randomizados, com um grupo comparativo.^5,6^ Foram realizadas análises de sensibilidade para avaliar a robustez dos achados, e uma metarregressão foi conduzida para explorar fontes potenciais de heterogeneidade.

### Análise estatística

Análise das diferenças médias agrupadas do tempo de cirurgia, tempo de *bypass*, suporte respiratório, e tempo de internação foi realizada usando o Open Meta.^5-9^ Nessa análise, nós incluímos todos os estudos que forneceram dados sobre tempo médio de intervenção e seu desvio padrão em um grupo com impressão em 3D em comparação a um grupo controle sem impressão em 3D. A análise agrupada utilizou a diferença média e a diferença média padrão pelo modelo de efeitos aleatórios DerSimonian-Laird. Para estudos que apresentaram mediana e intervalo do tempo de intervenção, nós estimamos a média e a variância conforme o proposto por Hozo et al.^7^ Para a proporção agrupada de mudanças na decisão cirúrgica, realizamos uma metanálise com braço único para o tamanho do efeito combinado usando o modelo de efeitos aleatórios de DerSimonian-Laird. Consideramos um intervalo de confiança de 95%, e um nível de significância estatística de 5%. A heterogeneidade foi estimada usando o Q-statistics (teste “Q de Cochran”). I2 e T2 foram fornecidos para quantificar inconsistências dos resultados entre os estudos como uma estimativa do desvio padrão da distribuição dos resultados. O viés de publicação não foi avaliado devido ao número limitado dos estudos incluídos. A sensibilidade da estimativa agrupada dos estudos individuais foi examinada usando a metanálise *leave-one-out*.

### Registro e protocolo

A revisão sistemática foi realizada seguindo-se o *Preferred Reporting Items for Systematic Reviews and Meta-Analyses* (PRISMA) (Apêndice 2).^8^ Além disso, o protocolo do estudo foi registrado no PROSPERO sob o número CRD42024543412.

## Resultados

### Seleção dos estudos

**Figura 1:**
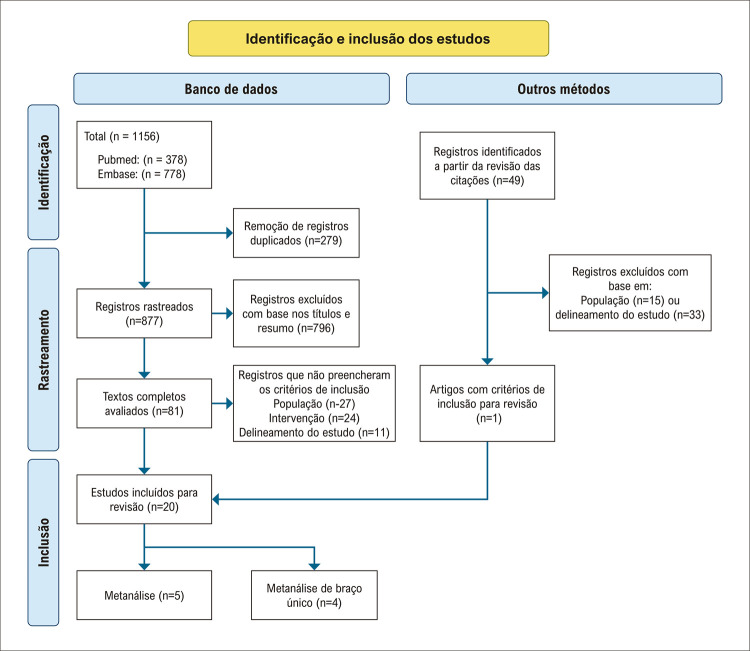
– Fluxograma PRISMA (Preferred Reporting Items for Systematic Reviews and Meta-Analyses) mostrando o fluxo das publicações pelo processo de revisão.

A busca na literatura gerou um total de 1156 resultados e 1069 estudos foram excluídos após rastreio por título e resumo, e remoção das duplicatas. Os 81 artigos restantes foram avaliados pelo texto completo, resultando em 20 artigos.^9-28^ Cinco artigos foram excluídos da metanálise por não haverem descrito o desfecho, e seis não eram estudo controlados. Assim, nove estudos foram selecionados para a metanálise (Figura 1).

### Características dos estudos

Cinco estudos compararam desfechos entre os grupos em que houve uso de impressão 3D no tratamento *versus* grupos submetidos a tratamentos convencionais, resultando em 219 pacientes. Sessenta e quatro casos foram operados com o auxílio de modelos impressos em 3D, e 153 pacientes foram submetidos à cirurgia sem auxílio da impressão em 3D. Quatro estudos incluíram dados para metanálise de braço único para avaliar a mudança no planejamento cirúrgico após a aplicação da impressão 3D, resultando em 75 pacientes.

A Tabela 1 resume as características dos estudos incluídos. A maioria dos estudos utilizou o *software* Mimics (Materialise) para reconstruir os modelos 3D a partir de imagens da TC e RM. A maioria das impressoras 3D usadas nos estudos eram de tecnologia de resina fotossensível.

**Tabela 1:** – Características basais dos estudos incluídos

Autores	Ano	Delineamento do estudo	N	Condição	Método de imagem	Software de segmentação	Modelo de impressora 3D	Materiais de impressão	Custos	Objetivo do estudo
Bhatla et al.^9^	2016	Série de casos	6	DSV, DVSVD	RM ou TC	Materialise Mimics	-	-	-	Planejamento perioperatório
Garekar et al.^10^	2016	Série de casos	5	DVSVD	RM ou TC	-	3D Systems Projet 660 pro full color	Filamento PLA	-	Planejamento perioperatório e educação
Han et al.^11^	2019	Estudo controlado	12 (6/6)	Interrupção do arco aórtico, DSV, DVSVD, hipoplasia do arco aórtico, atresia da aorta, coarctação da aorta	TC	-	Formlabs	Resina de fotopolímero	-	Desempenho
He et al.^12^	2019	Série de casos	5	CIA com defeito no seio venoso inferior	TC	Materialise Mimics	ZRapid SLA450 RAPID	Resina de fotopolímero	-	Planejamento perioperatório
Hoashi et al.^13^	2018	Série de casos	20	DVSVD, TGA, Interrupção do arco aórtico, TF	TC	-	SOUP2 600GS e SCS-8100	Resina de fotopolímero	Modelo/ 2000–3000 USD	Planejamento perioperatório
Kappanayil et al.^14^	2017	Série de casos	5	DVSVD complexa, dois pacientes com conexão atrioventricular entrecruzado (crisscross), TGVCC	RM ou TC	Materialise Mimics	-	Resina de fotopolímerou PLA	-	Planejamento perioperatório
Matsubara et al.^15^	2019	Estudo controlado	11 (4/7)	DAP	TC	Ziostation2 e OsiriX	UP Plus2 3D	Filamento de ABE	Modelo/ 90 USD	Planejamento e desempenho
Nam et al.^16^	2021	Estudo controlado	6	TF; estenose pulmonar complexa	TC	Materialise Mimics	Stratasys Object500 Connex	Resina fotossensível	Modelo/ 100 USD	Desempenho
Olivieri et al.^17^	2016	Série de casos	10	CCs múltiplas	RM ou TC	Materialise Mimics	Stratasys Object500 Connex	Resina fotossensível	Modelo/ 200 USD	Educação e conhecimento
Ryan et al.^18^	2018	Estudo controlado	146 (33/113)	Atresia pulmonar, TF, DVSVD, tronco arterioso, anéis vasculares, ventrículo único	RM ou TC	Geomagic e 3-matic	zPrinter 650	Resina fotossensível	-	Planejamento perioperatório, desempenho e aceitação
Shi et al.^19^	2021	Estudo controlado	23 (10/13)	Grupo com ventrículo equilibrado, grupo sem ventrículo equilibrado	TC	Materialise Mimics	BQ Witbox	Filamento PLA	-	Planejamento e desempenho
Sun et al.^20^	2017	Série de casos	5	Divertículo de Kommerell	TC	Medraw	Pangu V4.1	Filamento PLA	-	Planejamento perioperatório
Tiwari et al.^21^	2021	Estudo crossover	10	DVSVD com DSV e outras CCs de discordância ventriculoarterial	TC	Materialise Mimics	-	PLA ou filamento PLA	Modelo/ 350 USD	Planejamento perioperatório
Valverde et al.^22^	2017	Estudo crossover	40	Múltiplas CCs, predominantemente DVSVD DSV	RM ou TC	ITK-SNAP Software	BQ Witbox	Filamento de TPU	Modelo/ 300-500 USD	Planejamento perioperatório
Wang et al.^23^	2016	Série de casos	6	CIA com defeito de bordas	TC	Materialise Mimics	ZRapid SLA450 RAPID	Resina fotossensível	-	Planejamento perioperatório
Xu et al.^24^	2019	Série de casos	15	Múltiplas CCs	TC	Materialise Mimics	ISLA650	Resina fotossensível	-	Planejamento perioperatório
Xu et al.^25^	2019	Série de casos	17	Drenagem anômala total de veias pulmonares	TC	Materialise Mimics	ISLA650	Resina fotossensível	-	Planejamento perioperatório
Yan et al.^26^	2018	Série de casos	35	CIA com deficiência do óstio	TC	-	Objet350 Connex3	Resina fotossensível	1200–1300 USD/model	Treinamento e desempenho
Yan et al.^27^	2018	Série de casos	7	CIA com ausência de óstio da veia pulmonar direita	TC	Materialise Mimics	Objet350 Connex3	Resina fotossensível	-	Planejamento perioperatório
Zhao et al.^28^	2018	Estudo controlado	25 (8/17)	DVSVD	TC	Materialise Mimics	ZPrinter 650	Resina fotossensível	-	Planejamento e desempenho

CCs: cardiopatias congênitas; CIA: comunicação interatrial; TF: Tetralogia de Fallot; DVSVD: dupla via de saída do ventrículo direito; DSV: defeito do septo ventricular; PLA: ácido poliláctico; TGVCC: transposição dos grandes vasos congenitamente corrigida: ABE: acrilonitrila butadieno estireno; DAP: ducto arterioso patente; TPU: poliuretano termoplástico.

**Figura 2:**
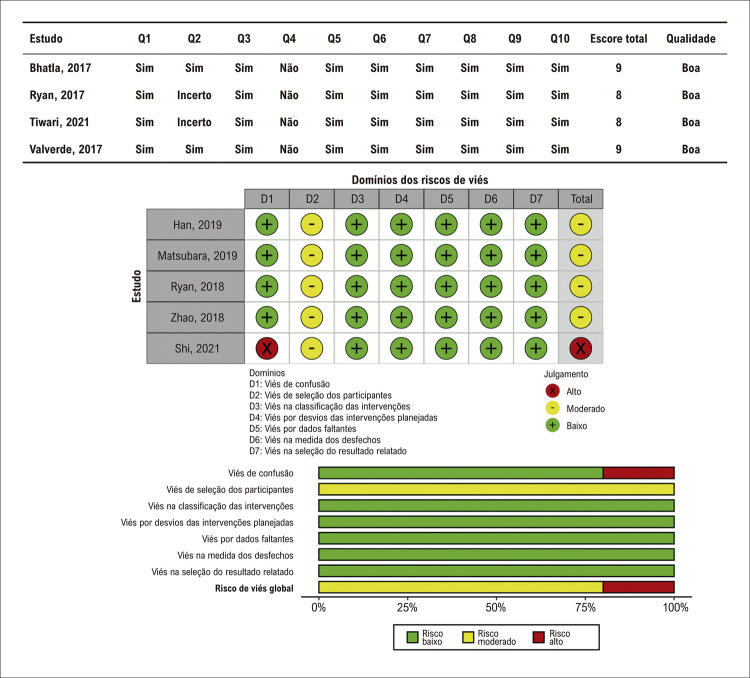
– Avaliação do risco de viés dos estudos incluído.

A avaliação do risco de viés nos estudos incluídos gerou algumas preocupações (Figura 2). Todos os estudos foram considerados com um risco moderado de viés no domínio “seleção dos participantes”. Conduzimos a metanálise apesar desse risco aumentado de viés, uma vez que não foi relatado se a seleção dos pacientes para o tratamento em que se utilizou impressão em 3D ocorreu antes ou após os exames de imagem. Ainda, nenhuma das séries de casos incluiu pacientes consecutivos, aumentando o risco de viés em nossos resultados.

### Mudança no plano cirúrgico

**Figura 3:**
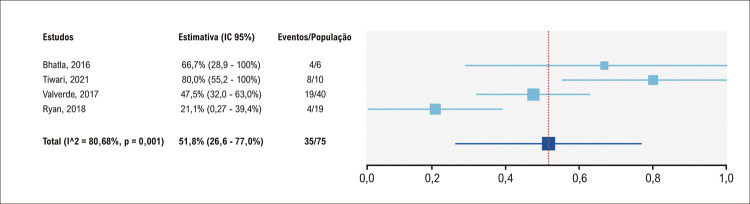
– Análise agrupada da mudança na decisão cirúrgica após interação com modelos impressos em 3D; IC: intervalo de confiança.

Quatro estudos incluíram dados sobre mudança nas decisões cirúrgicas. A Figura 3 resume a análise agrupada indicando uma taxa de 51,8% (IC95% 26,6-77,0%) de mudança no procedimento cirúrgico após o uso da impressão em 3D. Esses resultados indicam que modelos em 3D podem ser úteis no planejamento pré-operatório de casos complexos de CCs.

### Tempo total de cirurgia

**Figura 4:**
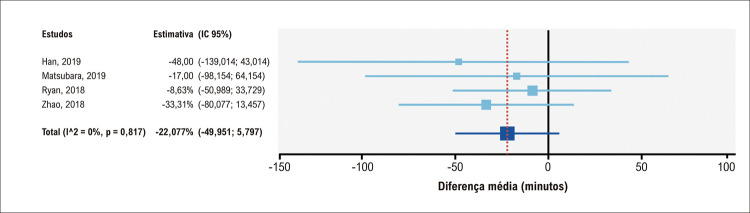
– Análise agrupada do tempo total de cirurgia no grupo submetido a tratamento com uso de impressão em 3D em comparação ao grupo submetido a tratamento convencional. IC: intervalo de confiança.

A maioria dos estudos controlados apresentavam dados sobre tempo médio de cirurgia. O grupo submetido a tratamento com modelos impressos em 3D apresentou uma média de tempo mais curta em comparação ao grupo submetido a tratamento convencional, com uma diferença média de 22,25 minutos, mas sem significância estatística; IC95% = 49,951–5,797 minutos (Figura 4).

### Desfechos secundários

**Figura 5:**
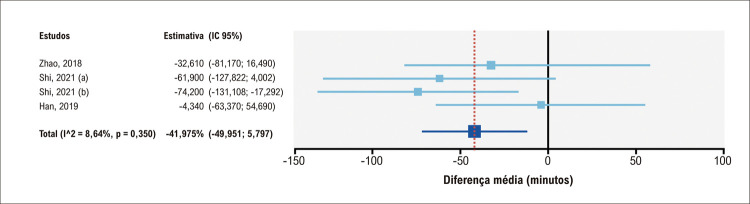
– Análise agrupada do tempo médio de bypass no grupo submetido a tratamento com uso de impressão em 3D em comparação ao grupo submetido a tratamento convencional. IC: intervalo de confiança.

Em comparação à terapia padrão sem impressão em 3D, a cirurgia guiada pela impressão em 3D em pacientes com CC apresentou uma redução significativa no tempo de *bypass*, com uma diferença média de 41,975 minutos; IC95% = 71,754 a -12,197 minutos (Figura 5). A heterogeneidade foi baixa (I^2^ = 8.64%), sem significância estatística, o que implica ausência de inconsistência dos resultados entre os estudos. Dois estudos foram incluídos no tempo de ventilação mecânica. E no tempo na unidade de terapia intensiva (Apêndices 4-7).

A Figura Central resume os principais achados deste artigo.

## Discussão

Nossa revisão demonstra alguns efeitos positivos da impressão em 3D personalizada sobre desfechos da cirurgia para CCs. Na literatura, a impressão em 3D foi predominantemente utilizada em defeitos cardíacos conotruncais e do septo atrial. As anomalias conotruncais estão geralmente associadas com uma geometria complexa, e os exames de imagem são uma ferramenta diagnóstica na avaliação no pré-operatório e no pós-operatório.^29^

A metanálise demonstra o impacto positivo potencial da tecnologia da impressão em 3D no tratamento cirúrgico da CC. Nas cirurgias com modelos de impressão em 3D, observou-se uma redução significativa no tempo do *bypass* e no tempo de intervenção em comparação à terapia padrão. A redução na duração cirúrgica não só aumenta a precisão e a eficiência dos procedimentos cirúrgicos como também minimiza os riscos associados com um tempo prolongado do *bypass*. Consequentemente, os pacientes podem se beneficiar de cirurgias com uma duração mais curta e recuperação potencialmente mais rápidas.

Revisões sistemáticas prévias relataram que modelos impressos em 3D fornecem aos cirurgiões uma representação precisa de anatomias cardíacas complexas, potencialmente levando a maior precisão cirúrgica e melhores desfechos dos pacientes.^30-32^ Uma metanálise de várias cirurgias cardíacas demonstrou uma redução no tempo total de cirurgia com uma diferença média padronizada 0,54 (IC95%: 0,13-0m95; p = 0,001), mas esse resultado apresentou alta heterogeneidade e incluiu outras cirurgias além de CCs.^31^ Além disso, os resultados agrupados demonstraram que modelos impressos em 3D apresentam um impacto sobre o planejamento no pré-operatório.^11,15,22^ Borracci et al.^33^demonstraram evidência similar em adultos com doença cardíaca não congênita, e seis dos 14 modelos redefiniram a abordagem cirúrgica.^33^

Modelos 3D podem ainda servir como ferramentas efetivas para explicar procedimentos cirúrgicos complexos a pacientes e seus familiares. Isso pode melhorar o entendimento da condição e da intervenção planejada, levando a um melhor consentimento informado e potencialmente reduzir a ansiedade. Um ensaio randomizado controlado demonstrou a utilidade de modelos impressos em 3D para o consentimento cirúrgico em defeitos perimembranosos.^34^

Além disso, a impressão em 3D ocupa um lugar mais bem estabelecido no treinamento e na educação. Modelos impressos em 3D podem ser valiosos para residentes em cirurgia e estudantes de medicina, uma vez que eles promovem uma experiência realista, prática, com CCs complexas, e melhores resultados educacionais e habilidades cirúrgicas.^15^ O uso da impressão em 3D nas cirurgias de CCs exemplifica as implicações positivas de integrar tecnologias inovadoras nas práticas médicas, levando a melhores desfechos dos pacientes, e estabelecendo novos padrões no tratamento cirúrgico.

Os resultados demonstram a utilidade da impressão em 3D em várias áreas. Suas implicações incluem o planejamento cirúrgico e a redução no tempo cirúrgico e na taxa de complicação. Acredita-se que uma das áreas mais promissoras da impressão em 3D é o treinamento cirúrgico, em que os cirurgiões conseguem realizar procedimentos complexos em uma zona sem riscos.^35^ Outra expectativa é a redução dos custos de produção e a maior acessibilidade ao equipamento. Existe, ainda, expectativa acerca da pesquisa sobre o uso de materiais impressos em 3D que mimetizam tecidos biológicos.^20^ Contudo, a eficiência da impressão em 3D depende do desenvolvimento das técnicas de segmentação e impressão, de maneira que elas possam ser incorporadas à prática médica posteriormente.^36^ Uma alternativa é a modelagem em 3D sem a impressão em 3D, que tem um custo mais baixo e pode ser usada em realidade virtualmente aumentada.

### Limitações

Nossa análise agrupada indicou que a impressão em 3D causa mudanças em 51,8% (IC95% 26,6-77,0% I^2^ = 80,7%) das decisões cirúrgicas em casos complexos de CCs. Deve-se considerar que a alta heterogeneidade em nossos resultados pode ser explicada por diferentes condições de CCs. Ainda, o possível viés de seleção e de relato pode haver superestimado esse resultado, uma vez que os estudos não incluíram pacientes consecutivos. Portanto, essa conclusão deve ser considerada com cuidado e representar melhor casos complexos das CC.

Os estudos incluídos variaram significativamente em termos das populações de paciente, tipos de CCs, tecnologias de impressão em 3D, e intervenções realizadas. Essa heterogeneidade pode dificultar a realização da metanálise ou de se tirar conclusões generalizadas. Porém, tal limitação somente influenciou os resultados agrupados de proporção, e a heterogeneidade nos demais resultados não foi significativa.^37^

Não foi avaliado viés de publicação devido ao número limitado de estudos em cada metanálise. Esta revisão pode ser afetada pelo viés de publicação, já que estudos com resultados negativos ou inconclusivos podem não ter sido publicados. Além disso, alguns estudos podem não haver fornecido informações completas sobre sua metodologia, resultados ou potenciais conflitos de interesse. Esse relato incompleto pode prejudicar a capacidade de se avaliar o risco de viés e a validade dos achados do estudo. Além disso, a interpretação dos modelos 3D e seu impacto sobre o planejamento cirúrgico pode influenciar os desfechos do estudo. Hussein et al.^38^ relataram que alguns cirurgiões jovens consideraram a tecnologia mais útil que cirurgiões experientes.

## Conclusão

Esta revisão sistemática destaca a evidência atual sobre o uso da impressão em 3D para as intervenções cirúrgicas de CCs. Esses modelos podem servir como uma ferramenta de planejamento pré-operatório e pode reduzir o tempo de cirurgia. Tais resultados devem ser confirmados em estudos com um número grande de casos e randomizados quanto à aplicação da tecnologia.
