# Supplementary material for: Impacto da Impressão em 3D nas Cirurgias de Cardiopatias Congênitas: Revisão Sistemática e Metanálise
Source: Arq Bras Cardiol. 2024 Nov 27;121(12):e20240430. [Article in Portuguese] doi: 10.36660/abc.20240430 (PMC11634304; doi:10.36660/abc.20240430)
Supplement: Translation

# Translation: Impact of 3D Printing on Cardiac Surgery in Congenital Heart Diseases: A Systematic Review and Meta-Analysis

**DOI:** 10.36660/abc.20240430i

**Authors:** Davi Shunji Yahiro, Mariana de Paula Cruz, Brenda Ficheira Coelho Ribeiro, Luiza Meireles Teixeira, Maria Fernanda Ribeiro Mendes de Oliveira, Aurea Lúcia Alves de Azevedo Grippa de Souza, Ana Flávia Malheiros Torbey, Juliana Serafim da Silveira, Claudio Tinoco Mesquita

**Affiliations:** 1 Brazil Universidade Federal Fluminense, Niterói, RJ – Brazil; 2 Brazil Pró-Cardíaco Hospital, Rio de Janeiro, RJ – Brazil

**Keywords:** Three-Dimensional Printing, Congenital Heart Defects, Preoperative Care, Precision Medicine

## Abstract

**Background:**

Congenital heart disease (CHD) poses significant challenges in surgical management due to the complexity of cardiac anatomy. Three-dimensional (3D) printing has emerged as a promising tool in preoperative planning, intraoperative guidance, and medical education for CHD surgeries.

**Objectives:**

We aimed to systematically review the literature on the utilization and benefits of 3D printing technology in CHD surgical interventions.

**Methods:**

A systematic search was conducted across PubMed and EMBASE for studies published up to February of 2024. We included controlled and uncontrolled studies investigating the surgical role of 3D printing in CHD patients. We conducted a single-arm meta-analysis estimating the proportion of change in treatment planning due to the use of 3D printed-models. Moreover, studies that compared 3D printing to conventional care were included into the meta-analysis. A p-value < 0.05 was considered statistically significant.

**Results:**

A total of 21 studies met the inclusion criteria, comprising 444 patients undergoing CHD surgeries with 3D printing assistance. Preoperative planning aided by 3D models led to changing surgical decisions in 35 of 75 cases (51.8%; 95% CI 26.6-77.0%, I2=80.68%, p=0.001) and reduced total operative time in 22.25 minutes in favor of the 3D printing group (95%CI 49.95; 5.80 min, I2=0%, p=0.817) but without statistical significance. Albeit in a smaller sample, other endpoints (mechanical ventilation and ICU time) demonstrated some benefit from the technology but without statistical significance.

**Conclusions:**

By providing personalized anatomical models, 3D printing may facilitate surgical planning and execution. More studies are needed to investigate the effects of 3D printing on reducing intervention, hospitalization, and mechanical ventilation times.

## Introduction

Cardiac anomalies are the most prevalent congenital malformations among live births in the world. In Brazil, it is estimated that nearly 25,757 new cases occur each year, and the southeast region presents the highest prevalence, with 10 new cases/1,000 live births. However, this number may be even higher due to the underreporting of cases of congenital heart disease.^1,2^

In many congenital heart diseases (CHD), a comprehensive analysis is necessary for the correct diagnosis and treatment as it can combine defects, adding complexity to each case. This requires an individualized and multidisciplinary treatment approach depending on their complexity. To plan an intervention, it is necessary to meticulously examine the anatomy of the structures. A meta-analysis demonstrated that the three-dimensional (3D) model of a CHD had a mean deviation of 0.04 mm, 95% CI (−0.16, 0.23) compared to the digital medical images.^3^Therefore, modeling and printing of 3D models from medical images may provide an auxiliary visualization of the specific anomaly.

3D models of any medical condition are possible by imaging exams. Computed tomography (CT) and magnetic resonance imaging (MRI) are the most reliable techniques to obtain data for constructing anatomical models. Before printing, the images need to be transformed into a digital model and divided into thin layers to be reconstructed by a 3D printer, forming the final piece. In recent years, 3D printing has emerged as a prominent technology within the field of medicine, offering versatile applications. Its use ranges from surgical planning, educational purposes, to effective communication strategies. The multifaceted nature of 3D printing has significantly advanced medical practices, enabling enhanced precision, efficiency, and outcomes.^4^

Despite the promising potential of 3D printing technology in improving the planning and execution of cardiac surgeries, particularly in patients with congenital heart diseases, there is a noticeable absence of robust data in this area. This lack of comprehensive and high-quality data limits our understanding of the true applicability and impact of 3D printing in this context. While preliminary studies and anecdotal evidence have suggested that 3D-printed heart models could provide surgeons with better preoperative insights and possibly improve surgical outcomes, the need for large-scale, systematic research is evident. Such studies would help quantify the benefits, optimize the use of this technology, and validate its efficacy and cost-effectiveness in the clinical setting. Until then, the full potential of 3D printing for planning cardiac surgeries remains largely unexplored and underutilized. Given the emergence in the medical environment, some systematic reviews assessed the impact of 3D printing in cardiovascular conditions.^3-6^However, none of them focused on CHD and the surgery intervention. In this regard, this study aims to evaluate and analyze the current applications of 3D printing in surgical interventions in CHDs.

## Methods

### Data source and Search Strategy

A systematic literature search was conducted across electronic databases including PubMed and Embase. The search strategy used a combination of words and terms related to 3D printing, CHDs, surgery, and interventions. Boolean operators and search filters were applied to ensure comprehensive coverage of relevant literature. The bibliography search was conducted in February of 2024, containing all works published until that date. The full search strategy is on Appendix 1. Additionally, manual searching of reference lists was employed to identify additional studies that may have been missed through electronic searches.

### Eligibility criteria and selection process

Two independent reviewers (DY and MC) screened titles and abstracts of retrieved citations to identify eligible studies. Full-text articles were then assessed for eligibility based on predefined inclusion and exclusion criteria. Studies were included if they evaluated the utility of 3D printing in surgical planning, performance, or prognosis of CHDs. Reviews, meta-analysis, letter, experimental studies, and case series with less than five participants were excluded from analysis. Any discrepancies between reviewers were resolved through discussion or consultation with a third reviewer.

### Data extraction

Data extraction was performed independently by two reviewers (DY, MC) using a standardized form. Extracted data included study design, sample size, 3D printer model, cost, imaging method, segmentation software, printing material, study purpose, condition in treatment, main findings such as change in surgery plan or time of surgery, and secondary outcomes: bypass time, mechanical ventilation, and length of hospitalization. We also collected information on change in surgery decision and ‘surgery time when available. Any disagreements between reviewers were resolved through consensus or consultation with a third reviewer.

### Risk of bias assessment

The quality of the included articles was appraised by two authors (MC and LT) using two different risk of bias tools – a JBI Critical Appraisal Checklist for Case Series for the retrospective studies without a comparison group and the ROBINS-I tool for prospective non-randomized studies with a comparison group.^5,6^ Sensitivity analyses were performed to evaluate the robustness of findings, and a meta-regression was conducted to explore potential sources of heterogeneity.

### Statistical analysis

Analysis of the pooled mean differences of surgical time, bypass time, respiratory support and length of hospital stay was performed using Open Meta Analyst.^5-9^ In this analysis, we included all studies that provided data on mean intervention time and its standard deviation in a group with 3D printing compared to a control group without 3D printing. The pooled analysis utilized the mean difference and standard mean difference by performing the DerSimonian-Laird random-effects model. For studies that provided median intervention time and its range, we estimated mean and variance according to Hozo et al.^7^ For the pooled proportion of changes in surgical decision, we performed a single-arm meta-analysis for the combined effect size using the DerSimonian-Laird random-effects model. We considered a 95% confidence interval, and 5% of statistical significance level. Heterogeneity was estimated by Q-statistics (“Cochran’s Q” test). I2 and T2 were provided to quantify inconsistencies of results across studies as an estimate of the standard deviation of the distribution of the results. Publication bias was not assessed because of the limited number of included studies. Sensitivity of pooled estimates of individual studies was examined using leave-one-out meta-analysis.

### Registration and protocol

This systematic review was performed in accordance with the Preferred Reporting Items for Systematic Reviews and Meta-Analyses (PRISMA) Appendix 2.^8^ The study protocol was registered in PROSPERO under the number of CRD42024543412.

## Results

### Study selection

The literature search yielded a total of 1,156 results, and 1069 studies were excluded after screening by title and abstract and removing duplicates. The remaining 81 articles were assessed for full text eligibility, obtaining 20 articles.^9-28^ Five articles were excluded from the meta-analysis because they did not report the outcome, and six were no controlled studies. Finally, nine studies were selected for the meta-analysis (Figure 1).

**Figure 1:** – Preferred Reporting Items for Systematic Reviews and Meta-Analyses (PRISMA) chart showing the flow of publications via the review process.

### Study characteristics

Five studies compared 3D printing groups to conventional care for outcomes, resulting in 219 patients. Sixty-four cases were operated on with the help of 3D-printed models, and 153 patients underwent surgery without 3D printing assistance. Four studies included data evaluated in the single-arm meta-analysis about changes in surgery planning after 3D printing application, resulting in 75 patients.

Table 1 summarizes the characteristics of included studies. Most studies used Mimics software (Materialise) to reconstruct the 3D models from CT or MRI scans. The photosensitive resin was the most common 3D printing technology observed in the studies.

**Table 1:** – Baseline characteristics of the included studies

Authors	Year	Study design	N	Condition	Imaging method	Segmentation software	3D Printer Model	Printing materials	Costs	Study purpose
Bhatla et al.^9^	2016	Case series	6	VSD, DORV	MRI or CT scans	Materialise Mimics	-	-	-	Perioperative planning
Garekar et al.^10^	2016	Case series	5	DORV	MRI or CT scans	-	3D Systems Projet 660 pro full color	PLA filament	-	Perioperative planning and Education
Han et al.^11^	2019	Control study	12 (6/6)	interrupted aortic arch, VSD, DORV, hypoplasia of aortic arch, aortic atresia, aortic coarctation	CT scans	-	Formlabs	Photopolymer resin	-	Performance
He et al.^12^	2019	Case series	5	ASD With an Inferior Sinus Venosus Defect	CT scans	Materialise Mimics	ZRapid SLA450 RAPID	Photosensitive Resin	-	Perioperative planning
Hoashi et al.^13^	2018	Case series	20	DORV, TGA, interrupted aortic arch, TOF	CT scans	-	SOUP2 600GS and SCS-8100	Photopolymer resin	2000–3000 USD/model	Perioperative planning
Kappanayil et al.^14^	2017	Case series	5	complex DORV, two patients with criss‐cross atrioventricular connections, CCTGA	MRI or CT scans	Materialise Mimics	-	Photopolymer resin or PLA	-	Perioperative planning
Matsubara et al.^15^	2019	Control study	11 (4/7)	PDA	CT scans	Ziostation2 and OsiriX	UP Plus2 3D printer	ABS filament	90 USD/model	Planning and Performance
Nam et al.^16^	2021	Control study	6	TOF; complex pulmonary stenosis	CT scans	Materialise Mimics	Stratasys Object500 Connex	Photosensitive Resin	100 USD/model	Performance
Olivieri et al.^17^	2016	Case series	10	Multiple CHD	MRI or CT scans	Materialise Mimics	Stratasys Object500 Connex	Photosensitive Resin	200 USD/model	Education and knowledge
Ryan et al.^18^	2018	Control study	146 (33/113)	Pulmonary atresia, TOF, DORV, truncus arteriosus, vascular rings, single ventricle	MRI or CT scans	Geomagic and 3-matic	zPrinter 650	Photosensitive Resin	-	Perioperatorive planning, performance and acceptance
Shi et al.^19^	2021	Control study	23 (10/13)	Balanced ventricular and Imbalanced ventricular group	CT scans	Materialise Mimics	BQ Witbox	PLA filament	-	Planning and performance
Sun et al.^20^	2017	Case series	5	Kommerell’s diverticulum	CT scans	Medraw	Pangu V4.1	PLA filament	-	Perioperative planning
Tiwari et al.^21^	2021	Crossover study	10	DORV with VSD and other CHDs of ventriculoarterial discordance	CT scans	Materialise Mimics	-	PLA or PVA filament	350 USD/model	Perioperative planning
Valverde et al.^22^	2017	Crossover study	40	Multiple CHD, predominatly DORV with VSD	MRI or CT scans	ITK-SNAP Software	BQ Witbox	TPU filament	300-500 USD/model	Perioperative planning
Wang et al.^23^	2016	Case series	6	ASD with rim deficiency	CT scans	Materialise Mimics	ZRapid SLA450 RAPID	Photosensitive Resin	-	Perioperative planning
Xu et al.^24^	2019	Case series	15	Multiple CHD	CT scans	Materialise Mimics	ISLA650	Photosensitive Resin	-	Perioperative planning
Xu et al.^25^	2019	Case series	17	Abnormal pulmonary venous drainage	CT scans	Materialise Mimics	ISLA650	Photosensitive Resin	-	Perioperative planning
Yan et al.^26^	2018	Case series	35	ASD with rim deficiency	CT scans	-	Objet350 Connex3	Photosensitive Resin	1200–1300 USD/model	Training and Performance
Yan et al.^27^	2018	Case series	7	ASD with no right pulmonary vein rim	CT scans	Materialise Mimics	Objet350 Connex3	Photosensitive Resin	-	Perioperative planning
Zhao et al.^28^	2018	Control study	25 (8/17)	DORV	CT scans	Materialise Mimics	ZPrinter 650	Photosensitive Resin	-	Planning and Performance

ASD: atrial septal defect; TOF: Tetralogy of Fallot; DORV: double outlet right ventricle; TGA: transposition of great arteries; VSD: ventricular septal defect; CCTGA: congenitally corrected transposition of great arteries; CT: computed tomography; MRI: magnetic resonance imaging; PLA: Polylactic Acid; ABS: Acrylonitrile Butadiene Styrene; PVA: Polyvinyl Alcohol; TPU: Thermoplastic Polyurethane; CHD: congenital heart disease; PDA: Patent ductus arteriosus.

### Risk of bias

The risk of bias assessment in the included studies resulted in some concerns (Figure 2). All studies were considered moderate risk of bias in the domain of “selection of participants”. We conducted the meta-analysis despite this increased risk of bias because it was not reported whether patient selection for 3D printing has occurred before or after the imaging exams. Also, none of the case series included consecutive patients, increasing the risk of bias in our results.

**Figure 2:** – Risk of Bias Assessment of the included studies.

### Change in surgery plan

Four studies included data on change in surgery decisions. Figure 3 summarizes the pooled analysis indicating a 51.8% (95% CI 26.6-77.0%) rate in changing the surgery procedure after the 3D printing use. These results indicate that 3D models may be helpful for preoperative planning in complex cases of CHD.

**Figure 3:** – Pooled analysis for change in surgery decision after 3D-printed model interaction. CI: confidence interval.

### Total surgery time

Most controlled studies had data on mean total surgery time. The group undergoing treatment with 3D-printed models had shorter mean operating times compared with the conventional group, with a mean difference of 22.25 minutes, but without statistical significance; 95% CI = 49.951–5.797 minutes (Figure 4).

**Figure 4:** – Pooled analysis of total surgery time on 3D printing group compared to conventional group. CI: confidence interval.

### Secondary outcomes

Compared to standard therapy without 3D printing, 3D printing-guided surgery in CHD patients had significant reduction on bypass time with a mean difference of 41.975 minutes; 95% CI = 71.754 to -12.197 minutes (Figure 5). Heterogenicity was low (I^2^ = 8.64%), with no statistical significance, which implies no inconsistencies of results across studies. Two studies were included in mechanical ventilation time, and intensive care unit time (Appendix 4-7).

**Figure 5:** – Pooled analysis of mean bypass time on 3D printing group compared to conventional group. CI: confidence interval.

The Central Illustration summarizes the main findings of this paper.

## Discussion

Our review demonstrates some positive effects of personalized 3D printing on surgical outcomes for CHDs. In the literature, 3D printing was found to be predominantly used in conotruncal pathologies and atrial septal defect. Conotruncal anomalies are often associated with a complex geometry, and imaging exams is an essential diagnostic tool in preoperative and postoperative assessment.^29^

This meta-analysis demonstrates the potential positive impact of 3D printing technology in the surgical treatment CHD. In surgeries with 3D-printed models, there was a significant reduction in bypass and intervention times compared to standard therapy. Reduction of surgical duration not only enhances the precision and efficiency of surgical procedures but also minimizes the risks associated with prolonged bypass time. Consequently, patients may benefit from shorter surgeries and potentially quicker recoveries.

Previous systematic reviews reported that 3D printed models can provide surgeons with accurate representation of complex cardiac anatomies, potentially leading to improved surgical precision and better patient outcomes.^30-32^ A meta-analysis of various cardiac surgeries demonstrated reduction in total operation time with a standardized mean difference of 0.54 (95% CI: 0.13-0.95, p = 0.001), but this result had high heterogeneity and included other surgeries besides CHD.^31^ Moreover, the pooled results demonstrated that 3D printed models have an impact on preoperative planning.^11,15,22^ Boracci et al.^33^ demonstrated similar evidence in adults with non-congenital heart disease, and six of the 14 models redefined surgical approach.^33^

3D models can also serve as effective tools for explaining complex surgical procedures to patients and their families. This can improve their understanding of the condition and the planned intervention, leading to better-informed consent and potentially reducing anxiety. A randomized control trial demonstrated the usefulness of 3D printed models for surgical consent in perimembranous ventricular septal defects.^34^

Moreover, 3D printing has a more established place in training and education. 3D printed models can be valuable for surgical residents and medical students, as they provide a realistic, hands-on experience with complex CHDs, enhancing educational outcomes and surgical skills.^15^ The use of 3D printing in CHD surgeries exemplifies the positive implications of integrating innovative technologies into medical practices, leading to improved patient outcomes, and setting new standards in surgical treatment.

The results demonstrated the usefulness of 3D printing in several areas. Its applications include surgical planning and reduction in the surgery time and in the complication rate. It is believed that one of the most promising areas of 3D printing is the surgical training, in which surgeons can perform complex procedures in a free risk zone.^35^ Another expectation is the reduction of production costs and higher accessibility of equipment. There also is expectation around the research on the use of 3D-printable materials that mimic biological tissues.^20^ However, the efficiency of 3D printing depends on development of segmentation and printing techniques, so they can be incorporated into medical practice thereafter.^36^ One option is 3D modeling without 3D printing, which is less costly and can be used in virtual augmented reality.

### Limitations

Our pooled analysis indicated that 3D printing changes 51.8% (95%CI 26.6-77.0% I^2^ = 80.68%) of the surgical decisions in complex cases of congenital heart diseases. It must be considered that the high heterogeneity in our results may be explained by different conditions of CHD. Also, the possible selection and reporting bias may have overestimated this result, as the studies did not include consecutive patients. Therefore, this conclusion must be taken with caution and be best representative for complex cases of CHDs.

The included studies varied significantly in terms of patient populations, types of CHDs, 3D printing technologies used, and interventions performed. This heterogeneity can make it difficult to perform a meta-analysis or draw generalized conclusions. However, this limitation only influenced the pooled proportion results, and heterogeneity in other results was not significant.^37^

Publication bias was not assessed due to the limited number of studies in each meta-analysis. This review may be affected by publication bias, since studies with negative or inconclusive results may have not been published. Moreover, some studies may have not provided complete information on their methodology, results, or potential conflicts of interest. This incomplete reporting can hinder the ability to assess the risk of bias and the validity of the study findings. Besides, the interpretation of 3D models and their impact on surgical planning can be subjective. Variations in surgeon expertise and experience with 3D printing can influence study outcomes. Hussein et al.^38^ reported that some young surgeons have considered the technology more helpful than experienced surgeons.

## Conclusion

This systematic review underscores the current evidence on the use of 3D printing for surgical interventions for CHD. These models may serve as a preoperative planning tool and may reduce operation time. These findings should be confirmed in studies with large numbers of cases and randomized for the application of technology.
